# Analyzing compound activity records and promiscuity degrees in light of publication statistics

**DOI:** 10.12688/f1000research.8792.2

**Published:** 2016-08-10

**Authors:** Ye Hu, Jürgen Bajorath

**Affiliations:** 1 Department of Life Science Informatics, B-IT, LIMES Program Unit Chemical Biology and Medicinal Chemistry, Rheinische Friedrich-Wilhelms-Universität, Bonn, D-53113, Germany

**Keywords:** ChEMBL, publications, bioactivity, compound, promiscuity

## Abstract

For the generation of contemporary databases of bioactive compounds, activity information is usually extracted from the scientific literature. However, when activity data are analyzed, source publications are typically no longer taken into consideration. Therefore, compound activity data selected from ChEMBL were traced back to thousands of original publications, activity records including compound, assay, and target information were systematically generated, and their distributions across the literature were determined. In addition, publications were categorized on the basis of activity records. Furthermore, compound promiscuity, defined as the ability of small molecules to specifically interact with multiple target proteins, was analyzed in light of publication statistics, thus adding another layer of information to promiscuity assessment. It was shown that the degree of compound promiscuity was not influenced by increasing numbers of source publications. Rather, most non-promiscuous as well as promiscuous compounds, regardless of their degree of promiscuity, originated from single publications, which emerged as a characteristic feature of the medicinal chemistry literature.

## Introduction

Given the large volumes of compounds and activity data that are becoming available in the public domain ^1^, mining of activity data can be expected to provide fresh insights into structure-activity relationships, compound distributions over current targets, or compound activity profiles. From activity data, target annotations of bioactive compounds can be systematically extracted and their current degree of promiscuity be determined ^2^. In this context, one can distinguish between “good” and “bad” promiscuity; the latter resulting from assay artifacts due to, for example, undesired compound pan-assay interference ^3, 4^ or aggregator ^5^ characteristics; the former from the ability of small molecules to specifically interact with multiple targets ^2^. A reliable assessment of so-defined good promiscuity usually depends on high data confidence ^1, 2^. The ability to specifically engage in interactions with multiple targets provides the molecular basis of polypharmacology associated with drugs or other bioactive compounds ^6– 8^. Therefore, a quantitative assessment of promiscuity is helpful to estimate the magnitude of cross-reactivity within the current spectrum of active compounds and targets and establish networks of ligand-target interactions for the prioritization of promiscuous vs. selective candidate compounds. The universe of all possible ligand-target interactions will most likely never be fully explored and data incompleteness ^9^ will -to a more or lesser extent- be omnipresent. However, currently accessible volumes of compound activity data are so large that we can expect to draw statistically meaningful trends from them, for example, in the study of structure-activity relationships and activity cliffs or compound activity profiles. Most recent analyses of compound promiscuity on the basis of high-confidence activity data from medicinal chemistry have revealed that compounds covering the current spectrum of thousands of targets are on average active against one or two targets ^10^. This low degree of detectable promiscuity was found to be essentially stable over time, especially during periods of exponential compound data growth over the past decade ^11^. Even the currently most extensively assayed compounds extracted from the PubChem BioAssay database ^12^, tested against hundreds of targets, were on average only active against two or three targets ^13^, although one might anticipate particularly high degrees of compound promiscuity in screening assays. Given the large numbers of assay results available for these screening hits, the analysis provides an upper-level estimate of compound promiscuity. The results further support a more conservative view of promiscuity among bioactive compounds. It is noted that compound promiscuity was found to be consistently lower than promiscuity of approved drugs, with a mean of about four targets per drug ^14^, again assessed on the basis of high-confidence activity data. These findings give rise to speculations concerning possible reasons for the higher degree of drug promiscuity ^13^.

One might anticipate that compounds annotated with a single target are only reported in a single publication. Furthermore, one might also assume that compounds active against large numbers of targets are often extensively tested by different research groups and thus reported in many different publications. Therefore, in our current study, we add an additional layer of information to the analysis of compound activity profiles and promiscuity by tracing activity annotations back to source publications and determining their distribution over the literature. Although elaborate databases such as ChEMBL ^15, 16^, the major public repository for compounds and activity data from medicinal chemistry, largely rely on the extraction of data from the literature, publication information has thus far not been taken into consideration when analyzing activity data on a large scale. Therefore, we have systematically generated compound activity records from original publications and also analyzed promiscuity in relation to publication statistics.

## Materials and methods

### Data selection and curation

From the latest version of ChEMBL ^15, 16^ (release 21), compounds were assembled for which direct interactions (i.e. assay relationship type “D”) with single human protein targets at the highest confidence level (assay confidence score “9”) and defined potency measurements (K _i_ and/or IC _50_ values) were reported. All approximate measurements (e.g. “>”, “<”, or “~”) were disregarded. These compounds and their activity records were designated “set 1” and represented a high-confidence data set according to previously established confidence criteria ^17^. For comparison, a “set 2” was collected consisting of compounds with defined potency values (excluding approximate measurements) for single human protein targets. Hence, in this case, no assay type and confidence criteria were applied. In both cases, only activity measurements were considered that were reported in original publications and all of these publication records were collected.

### Data organization

Compound data sets 1 and 2 were further organized and analyzed on the basis of:

***Publications.*** Compounds and activity data were assigned to individual publications and grouped by publications using compounds, assays, and targets as criteria.

***Activity records.*** All individual compound-target combinations were determined to generate “activity records”. A compound might be tested against the same target in different assays reported in a single or multiple publications. In addition, potency values might vary across different assays and publications or might be referenced in other publications. Therefore, for each activity record representing a unique compound-target combination, all corresponding publications and potency values were collected and added to the record.

***Compounds.*** Publications and activity data were also grouped by compounds, leading to the definition of four subsets including compounds active against

(A) a single target reported in a single publication;

(B) a single target reported in more than five publications;

(C) more than five targets reported in a single publication;

(D) more than five targets reported in more than five publications.

The selection of cut offs, i.e. one and five targets, was based on the previous observations ^10^ that the majority of bioactive compounds were active against a single target and only approximately 1% of the compounds interacted with more than five targets. Therefore, a promiscuity degree of five (targets) would refer to highly promiscuous compounds. The same cut offs were applied to the number of associated publications.

### Promiscuity

For sets 1 and 2, the degree of promiscuity of a compound was defined as the number of targets it was reported to be active against ^2^. Promiscuity degrees were determined and analyzed in light of publication statistics.

## Results and discussion

### Activity data from the medicinal chemistry literature

Given our data selection and curation criteria described above, set 1 contained 168,208 unique compounds that were tested in 31,578 assays against 1566 human targets, as reported in Table 1. These activity data were reported in 11,213 publications from 70 different medicinal chemistry journals. Table 2 lists the top-ranked journals where most of these publications appeared. These eight journals published ~97% of the qualifying papers. In addition, a total of 318,570 potency measurements were available and associated with 257,138 unique activity records, which were defined as individual compound-target entries containing all associated publications and qualifying potency measurements. In addition, set 2 comprised 293,736 compounds yielding 621,704 potency measurements against 2170 human targets ( Table 1), which were reported in 19,528 publications from 90 journals ( Table 1 and Table 2). A total of 471,442 unique activity records were obtained.

**Table 1.** Data sets.

Number of		Set 1	Set 2
**Compounds**		168,208	293,736
**Assays**		31,578	66,336
**Targets**		1566	2170
**Activity records** **(compound-target combinations)**		257,138	471,442
**Potency measurements**		318,570	621,704
**Publications**	**All**	11,213	19,528
	**Single assay/** **target**	4449 (39.7%)	6440 (33.0%)
	**Multiple assays/** **single target**	1483 (13.2%)	3268 (16.7%)
	**Multiple assays/** **targets**	5281 (47.1%)	9820 (50.3%)

For sets 1 and 2, the number of compounds, assays, targets, activity records, and potency measurements is given. In addition, for both sets, the total number of publications and subsets reporting activity values from a single assay, multiple assays for the same target, or multiple assays for different targets are provided.

**Table 2.** Journals with largest numbers of source publications.

Journal	Number of publications	
	Set 1	Set 2
Bioorg. Med. Chem. Lett.	4456	8218
J. Med. Chem.	3417	6717
Bioorg. Med. Chem.	1424	1904
Eur. J. Med. Chem.	689	875
ACS Med. Chem. Lett.	419	547
J. Nat. Prod.	200	364
MedChemComm	186	212
Med. Chem. Res.	111	121

The top eight journals with more than 100 qualifying source publications for sets 1 and 2 are listed.

### Assays, targets and compounds in original publications

**Figure 1.**
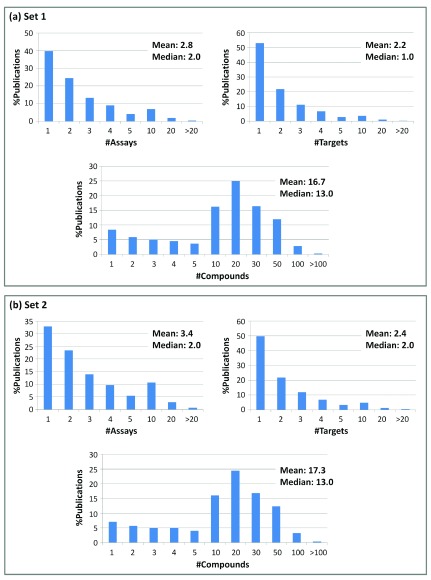
Distribution of assays, targets, and compounds in original publications. Histograms monitor the percentages of publications reporting increasing numbers of assays, targets, and compounds for ( **a**) set 1 and ( **b**) set 2, respectively. In addition, the mean and median values are provided.

Table 1 also reports the distribution of assays and targets over source publications. Of the nearly 11,000 papers associated with set 1, 4449 (~40%) and 1483 (~13%) reported activity data derived from a single assay and multiple assays for an individual target, respectively. The remaining ~47% of the publications reported activity from multiple assays for two or more targets. Similar observations were made for set 2 ( Table 1). Publications were further organized with respect to increasing numbers of assays, targets, and active compounds ( Figure 1). The majority of publications of sets 1 and 2 reported one or two assays for one or two targets, while ~9% (set 1) and ~14% (set 2) of the papers contained results for more than five assays. In addition, ~5% (set 1) and ~6% (set 2) of the publications reported activity data for more than five targets. On average, a set 1 and set 2 publication reported 2.8 and 3.4 assays for 2.2 and 2.4 targets and 16.7 and 17.3 active compounds, respectively ( Figure 1). Hence, assay, compound, and target statistics were very similar for both sets.

### Activity records from source publications

**Figure 2.**
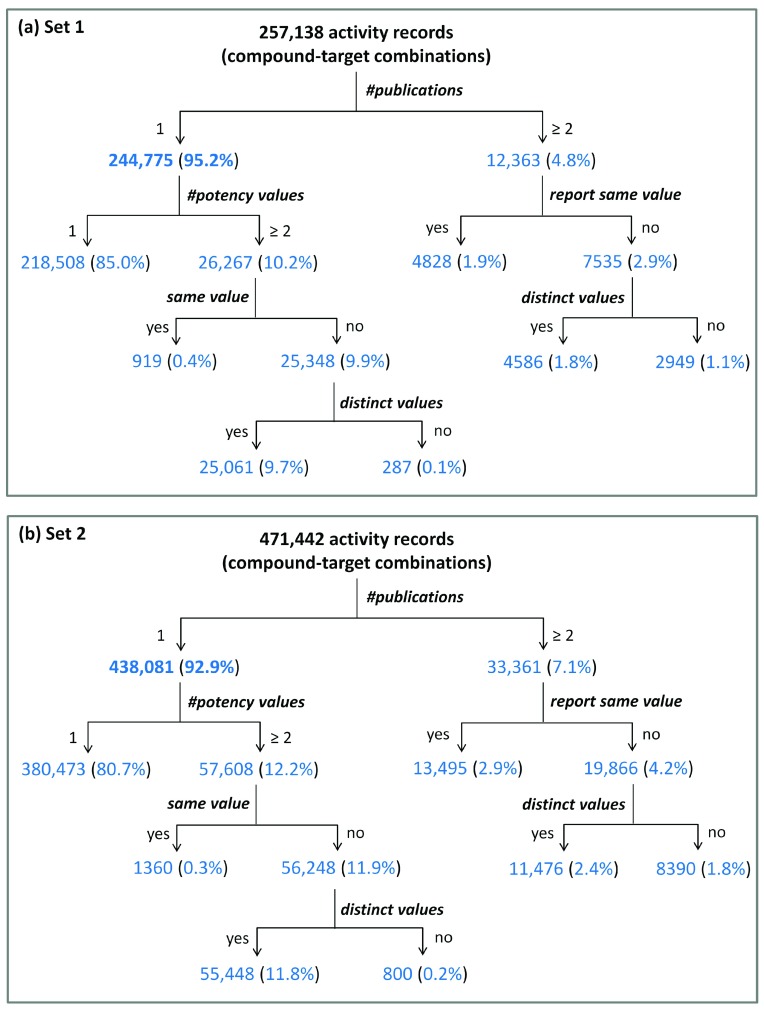
Classification of activity records. Activity records from ( **a**) set 1 and ( **b**) set 2 are classified using a decision tree structure.

From set 1 and set 2 publications, a total of 257,138 and 471,442 unique activity records were extracted, respectively. These activity records were classified according to the number of publications from which they originated and the number of different potency values that were reported for each compound-target combination ( Figure 2).

Figure 2a shows that ~95% (244,775) of the set 1 activity records originated from a single publication. Most of these activity records (218,508) were associated with a single potency value. In addition, for 26,267 records, two or more potency values were available that mostly differed. Varying potency values typically resulted from different experiments. Of the 12,363 activity records originating from two or more publications, 7535 had varying potency values, whereas 4828 were associated with multiple instances of the same value, which was likely referenced from a previous publication. A similar distribution of activity records was observed for set 2 ( Figure 2b). Taken together, the results revealed that more than 90% of all activity records resulted from a single publication most of which appeared between 2006 and 2014.

### Activity records covering many publications

**Figure 3.**
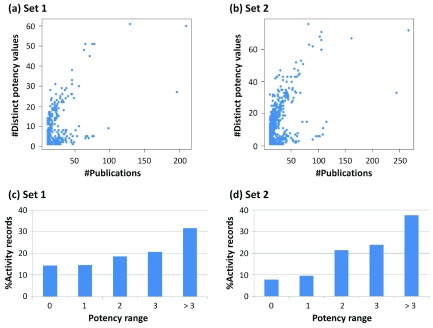
Activity records covering many publications. For ( **a**) 328 (set 1) and ( **b**) 632 (set 2) activity records (compound-target combinations) originating from more than 10 publications, the number of publications is plotted vs. the number of different potency values that were reported. In addition, in ( **c**) (set 1) and ( **d**) (set 2), the percentages of activity records covering increasing logarithmic potency ranges are given, e.g. “> 3” refers to a potency range of more than three orders of magnitude.

**Figure 4.**
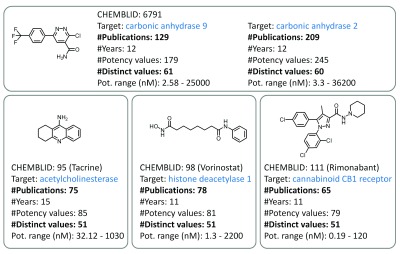
Extensively tested compounds. Shown are four compounds from set 1, which were tested against a given target in many publications and for which the largest numbers of distinct potency values were reported. Publication and potency value statistics are provided. CHEMBLID gives the compound identifier in ChEMBL. In addition, if available, compound or drug names are given in parentheses.

Small subsets of 328 (set 1) and 632 (set 2) activity records originated from more than 10 publications. Figure 3a (set 1) and Figure 3b (set 2) report the relationships between the number of publications and distinct potency values associated with these records. Up to 20 different potency values were frequently observed, which often spanned an unexpectedly large potency range of two or more orders of magnitude, as shown Figure 3c (set 1) and Figure 3d (set 2). Figure 4 shows exemplary compounds from such activity records, which further illustrate these findings. For example, the compound at the top was involved in two activity records with isoforms of carbonic anhydrase, a “classical” target, which were associated with 129 and 209 publications, respectively, appearing over a period of 12 years. In both instances, the range of 60 or 61 distinct potency values spanned nearly four orders of magnitude, revealing very large variations of experimental assessments.

### Promiscuity degrees and publication frequency

For each of 168,208 and 293,736 unique compounds from sets 1 and 2, the degree of promiscuity was determined, as reported in Table 3, revealing comparable distributions over degree intervals. Consistent with previous findings, the majority of bioactive compounds were found to interact with a single target ^10^. The mean degree of promiscuity was 1.5 for set 1 and 1.6 for set 2 and the median degree was 1.0 in both cases, also consistent with earlier findings ^10^. However, the low degree of promiscuity detected for set 2 was rather surprising because in this case, assay type and confidence criteria were not applied. The only requirement for set 2 compounds was the availability of clearly specified potency values for human protein targets, which resulted in promiscuity degrees very similar to set 1 having higher data confidence. These findings indicated that the requirement of explicit potency values alone limited the number of target annotations, although potency values for the same target often differed in their magnitude. Table 4 reports the publication frequency of all compounds in sets 1 and 2. Consistent with the results obtained for activity records, most of the compounds were only found in one publication, regardless of whether one or more targets were investigated.

**Table 3.** Compound promiscuity.

Number of targets (promiscuity degree)	Number of compounds (%)	
	Set 1	Set 2
**1**	117,253 (69.7%)	197,846 (67.4%)
**2**	30,457 (18.1)	57,466 (19.6)
**3**	12,092 (7.2)	22,308 (7.6)
**4**	5214 (3.1)	9172 (3.1)
**5**	1514 (0.9)	3295 (1.1)
**6–10**	1368 (0.8)	2892 (0.1)
**11–20**	280 (0.2)	621 (0.2)
**> 20**	30 (0.02%)	136 (0.05%)

For set 1 and set 2, the number (percentage) of compounds with increasing numbers of confirmed targets (degrees of promiscuity) is reported.

**Table 4.** Publication statistics.

Number of publications	Number of compounds (%)	
	Set 1	Set 2
**1**	158,995 (94.5%)	270,929 (92.2%)
**2**	7054 (4.2)	17,174 (5.9)
**3**	991 (0.6)	3023 (1.0)
**4**	398 (0.2)	921 (0.3)
**5**	200 (0.1)	473 (0.2)
**6–10**	327 (0.2)	719 (0.2)
**11–20**	146 (0.1)	300 (0.1)
**> 20**	97 (0.06)	197 (0.07)

For set 1 and set 2, the number (percentage) of active compounds reported in increasing numbers of publications is given.

### Promiscuity on the basis of source publications

**Figure 5.**
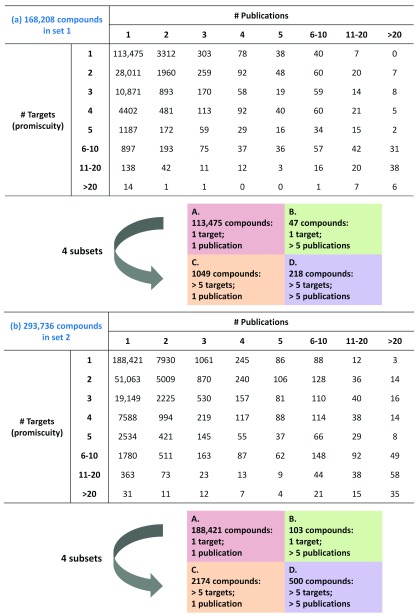
Compound promiscuity vs. publication frequency. In ( **a**) (set 1) and ( **b**) (set 2), compounds with increasing numbers of targets (top to bottom) reported in increasing numbers of publications (left to right) are given in a matrix format. In addition, four compound subsets (A–D) are defined.

**Figure 6.**
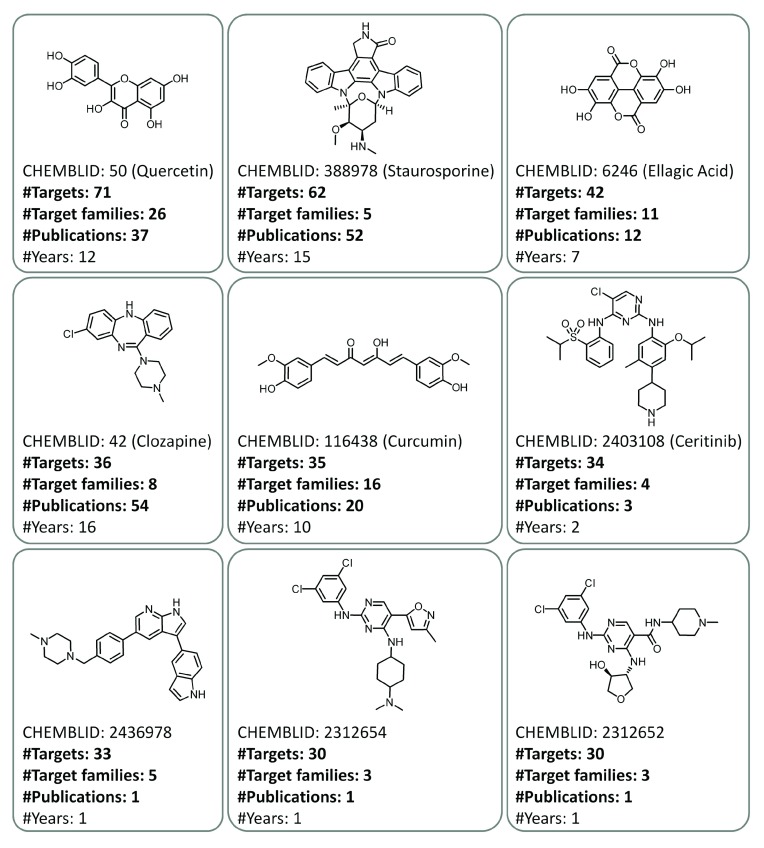
Highly promiscuous compounds. Shown are nine compounds displaying the largest degrees of promiscuity in set 1. Publication statistics are provided. In addition, if available, compound or drug names are given in parentheses.

Promiscuity was also assessed by directly focusing on source publications instead of activity records. The results are summarized in Figure 5. For both set 1 ( Figure 5a) and set 2 ( Figure 5b), target annotations of compounds across all promiscuity degrees mostly originated from a single publication, although multiple publications also contributed in many instances. There was no detectable correlation between promiscuity degrees and the number of source publications. Four subsets of compounds (A–D) were defined covering different ranges of promiscuity degrees and source publications. In set 1 ( Figure 5a), 113,475 (67.5%; subset A) and 47 (0.03%; subset B) compounds with a promiscuity degree of 1 originated from a single and more than five publications, respectively. In addition, 1049 (0.6%; subset C) and 218 (0.1%; subset D) compounds with a promiscuity degree >5 originated from a single and more than five publications, respectively. Thus, activity data characterizing most of the highly promiscuous compounds were also reported in a single publication. Equivalent observations were made for compounds in set 2 ( Figure 5b). The nine most promiscuous compounds from set 1 are shown in Figure 6. These compounds were annotated with 30 to 71 targets belonging to three to 26 families reported in one to more than 50 publications. Overall more than 86% of promiscuous compounds originated from single publications and there was no relationship between the degree of promiscuity and increasing numbers of source publications. Hence, current degrees of compound promiscuity could not be attributed to publication statistics and cumulative effects.

## Conclusions

In this study, compound activity records were systematically extracted from original publications and their distribution was analyzed. Furthermore, publications were classified on the basis of activity records. For given compound-target combinations, potency value ranges from different experiments were often unexpectedly large, although only well-defined potency measurements were considered (K _i_ or IC _50_ values). At the same time, the exclusive consideration of numerically explicitly defined potency measurements for human targets led to essentially the same promiscuity estimates as the use of higher-confidence activity data taking assay type and confidence criteria into account. For promiscuity exploration on the basis of compound activity data, the immediate focus on source publications added an as of yet missing piece to the analysis puzzle. Since the majority of promiscuous compounds, regardless of their degree of promiscuity, were traced back to single publications, there was not notable bias due to publication frequency and statistics. Negative results are typically not reported in the scientific literature when known active compounds are re-tested on other potential targets. Therefore, test frequency does only influence publication frequency if positive results are obtained. Potential evidence for such effects is currently only available for very small numbers of active compounds, leading to an overall consistent picture of low promiscuity among bioactive compounds, consistent with earlier investigations.

## Data availability

The data referenced by this article are under copyright with the following copyright statement: Copyright: © 2016 Hu Y and Bajorath J

The data selection criteria specified herein make it possible to directly reproduce all data sets from ChEMBL version 21. However the data generated for this study are also made freely available on Zenodo: Compound activity records associated with original publications in ChEMBL 21, doi: 10.5281/zenodo.51688 ^18^. The organization of data sets and calculation of promiscuity degrees were carried out using in-house Perl scripts that can be easily reproduced by following the description given in the Methods section.
