# Supplementary material for: Analyzing compound activity records and promiscuity degrees in light of publication statistics
Source: F1000Res. 2016 Aug 10;5:Chem Inf Sci-1227. Originally published 2016 Jun 6. [Version 2] doi: 10.12688/f1000research.8792.2 (PMC4916991; doi:10.12688/f1000research.8792.2)
Supplement: Peer review history

# Peer review: Referee response for version 2

**DOI:** 10.5256/f1000research.10142.r15579

**Published:** 2016-08-11

**Authors:** Kimito Funatsu

**Affiliations:** 1 Department of Chemical System Engineering, School of Engineering, The University of Tokyo, Tokyo, Japan

I have read this submission. I believe that I have an appropriate level of expertise to confirm that it is of an acceptable scientific standard.

# Peer review: Referee response for version 1

**DOI:** 10.5256/f1000research.9463.r14466

**Published:** 2016-06-20

**Authors:** Hans Matter

**Affiliations:** 1 Sanofi R&D, Sanofi-Aventis Deutschland GmbH , Frankfurt, Germany

This interesting contribution by Bajorath *et al*. extends previous database analysis work in order to identify and annotate promiscuous compounds. The authors extract activity information from public databases like ChEMBL and then trace back this information to the original primary scientific literature. It has been often documented that multiple compounds interact not only with single targets, but sometimes with many desirable and / or undesirable targets (off-targets). Further analysis of these polypharmacology findings is of great utility in understanding drug profiles and striving for the design of molecular with better overall profiles.

 Additional test campaigns after identification of bioactive compounds often reveal additional target-ligand interactions, both on undesirable ADMET targets (hERG, CYP, transporters) and selectivity off-targets (GPCRs, neighbouring proteins). However, these campaigns are expensive and will only systematically be conducted for molecules with interesting biological data and overall profile. Therefore for most compounds in the primary literature, only a single assay data point is reported to discuss the SAR of a particular series. It is very unlikely that this situation will significantly change in the near future.

 The report title and abstract cover the content well. The chemoinformatics approach is well conducted, clearly described and can most likely be reproduced by others. The results are presented in a clear and interesting way and capture the interest of F1000Research readers. The large dataset for this analysis was made publically available. The authors might also want to mention, whether software tools and subroutines from their study are available. Therefore this contribution is an essential view on available data for polypharmacology studies and should be indexed in its present form.

 I suggest that chemical structures displayed in figures 4 and 6 should be annotated with their trivial naves or drug names, if available. Furthermore groupings of the targets for compounds in both figures by target families might be instructive to see, whether compounds like staurosporine or flavones have only been tested for kinases or in a much broader manner.

 Furthermore implications of these results should be clearly discussed in the paper. This could also prompt for additional suggestions and guidelines on conducting in-silico polypharmacology studies on these sparse data-matrices.

I have read this submission. I believe that I have an appropriate level of expertise to confirm that it is of an acceptable scientific standard.

# Peer review: Referee response for version 1

**DOI:** 10.5256/f1000research.9463.r14194

**Published:** 2016-06-13

**Authors:** Kimito Funatsu

**Affiliations:** 1 Department of Chemical System Engineering, School of Engineering, The University of Tokyo, Tokyo, Japan

This manuscript brings a great amount of investigative work, aiming to provide further insight regarding activity data and promiscuity degrees to publication statistics. The work was well conducted and the data was presented in a way that is rather informative for the readers. Besides some grammar mishaps and a few points that need clarification, I recommend this work to be accepted for indexation once the following considerations are addressed.

- The relation between promiscuity, or activity data, and single publications is relevant and the conclusions states that well. I believe, however, that this work lacks mentioning the full impact of such discovery. Some discussion is presented, but certain aspects should be highlighted more. How does this new development fit with previous investigations? Why is this conclusion important for those working with activity data and promiscuity?
- p.2 When presenting data organization, why did the author decide to group compounds based on 1 and 5 targets? Any particular reason for setting multiple targets as 5, and not 4, 6, etc.?
- p.2 “a conservative of promiscuity”. I understand what do you mean by it, but it should be better phrased for clarity’s sake.

I have read this submission. I believe that I have an appropriate level of expertise to confirm that it is of an acceptable scientific standard.
